# Synergism between 5-fluorouracil and N-methylformamide in HT29 human colon cancer line.

**DOI:** 10.1038/bjc.1990.82

**Published:** 1990-03

**Authors:** N. Laudonio, G. Zupi, E. Erba, C. Leonetti, M. D'Incalci

**Affiliations:** Instituto di Ricerche Farmacologiche Mario Negri, Milan, Italy.

## Abstract

In HT29 cells 5-fluorouracil (5FU) cytotoxicity is enhanced by subsequent incubation of cells in medium containing 1% N-methylformamide (NMF). This enhancement does not appear to be related to differences in the repair of 5FU-induced DNA damage. It is proposed that the inhibition of DNA synthesis by NMF (that is reversible and does not result in any detectable toxicity) becomes a lethal event in a cell in which DNA synthesis has already been altered by 5FU exposure. The synergism is sequence dependent (i.e. it does not occur when NMF is given before 5FU) and specific for some cell types as shown by the fact that no synergism was found in L1210 mouse leukaemia cells. In nude mice transplanted s.c. with HT29 cells daily 5FU treatment (for 5 days) followed by daily NMF treatment (for 10 days) caused much greater inhibition of tumour growth than either drug alone or the same combination given in the opposite order (NMF then 5FU). These results, if confirmed on other human colon tumours, could be of clinical interest as a means of increasing the therapeutic efficacy of 5FU in patients with colon cancer.


Br. J. Cancer (1990), 61, 377-381 © Macmillan Press Ltd., 1990

### Synergism between 5-fluorouracil and N-methylformamide in HT29 human colon cancer line

N. Laudonio[1,2], G. Zupi[2], E. Erba[1], C. Leonetti[2] & M. D'Incalci[1]

[1]*Istituto di Ricerche Farmacologiche 'Mario Negri', Via Eritrea 62, 20157 Milan; and* [2]*Istituto Regina Elena per lo Studio e la Cura dei Tumori, V.le Regina Elena 291, 00161 Rome, Italy.*

**Summary** In HT29 cells 5-fluorouracil (5FU) cytotoxicity is enhanced by subsequent incubation of cells in medium containing 1% N-methylformamide (NMF). This enhancement does not appear to be related to differences in the repair of 5FU-induced DNA damage. It is proposed that the inhibition of DNA synthesis by NMF (that is reversible and does not result in any detectable toxicity) becomes a lethal event in a cell in which DNA synthesis has already been altered by 5FU exposure. The synergism is sequence dependent (i.e. it does not occur when NMF is given before 5FU) and specific for some cell types as shown by the fact that no synergism was found in L1210 mouse leukaemia cells. In nude mice transplanted s.c. with HT29 cells daily 5FU treatment (for 5 days) followed by daily NMF treatment (for 10 days) caused much greater inhibition of tumour growth than either drug alone or the same combination given in the opposite order (NMF then 5FU). These results, if confirmed on other human colon tumours, could be of clinical interest as a means of increasing the therapeutic efficacy of 5FU in patients with colon cancer.

The polar solvent N-methylformamide (NMF) has recently been reported to induce the expression of a better differentiated or less malignant phenotype in both rodent and human tumour cells (Langdon & Hickman, 1987). Cellular alterations associated with NMF-mediated induction of differentiation include changes in morphology, clonogenicity, tumorigenicity and cell culture doubling time (Zupi *et al.*, 1988), which ultimately result in a lower tumour aggressiveness. NMF-treated cells revert to the original phenotype upon removal of the inducer, but the maturational treatment has been associated with increased tumour cell sensitivity to certain cytotoxic agents, such as X-rays, cisplatinum and bleomycin (Dexter *et al.*, 1984; Harpur *et al.*, 1986; Langdon *et al.*, 1985; Leith *et al.*, 1985, 1986). It has been proposed that the sequential use of cytotoxic and differentiating agents might improve anti-cancer therapy by preventing or slowing tumour cell proliferation and phenotypic diversification (Lotan & Nicolson, 1988).

In the human colon cancer cell line HT29 we have already studied the lethal effects of the combination of NMF and 5-fluorouracil (5FU) (Zupi *et al.*, 1988). 5FU cytotoxicity was potentiated by subsequent exposure of cells to a non-toxic dose of NMF (1%), but when the two compounds were given in the opposite sequence there was no synergism. The enhanced cytotoxicity of the sequence 5FU to NMF could be related to the NMF-treated cells being less able to recover from the sublethal damage produced by the anti-metabolite.

In order to shed some light on the mechanism of this potentiation we designed a study to assess the DNA damage and alteration of macromolecule synthesis caused by 5FU alone or 5FU followed by NMF on HT29 cells.

### Materials and methods

### *Cell cultures*

The HT29 colon adenocarcinoma cell line was maintained as monolayer culture in RPMI 1640 medium supplemented with 10% FCS, L-glutamine and antibiotics. Cells were harvested using 0.02% EDTA-0.05% trypsin solution. L1210 murine leukaemia cells were grown as a suspension culture in RPMI 1640 medium supplemented with 10% heat-inactivated FCS, L-glutamine, 20 mM Hepes and 10 μM β-mercaptoethanol. Both cell lines were maintained at 37°C in a humidified 5% $CO_2$: 95% air atmosphere. All products were purchased from GIBCO.

### *Chemicals and drugs*

N-Methylformamide (NMF, Sigma M2769) was diluted in normal growth medium. 5-Fluorouracil (5FU, Roche S.p.a., Milano) as supplied for clinical use, was diluted in saline and then in culture medium to the desired final concentration. $^{14}$C-thymidine (spec.act. 61 mCi mmol$^{-1}$), methyl-$^3$H-thymidine (spec.act. 70-85 Ci mmol$^{-1}$), 5-$^3$H-uridine (spec.act. 25-30 Ci mmol$^{-1}$) and L-4,5-$^3$H-leucine (spec.act. 45-70 Ci mmol$^{-1}$) were obtained from Amersham International plc, England.

### *Flow cytometry*

For cell cycle studies, cells were stained with 2 ml of a solution composed of the double-stranded nucleic acid probe propidium iodide (Calbiochem Boehring Co., St Louis, USA), 50 μg ml$^{-1}$ in 0.1% sodium citrate. Fifteen μl of RNase (Calbiochem) 0.5 mg ml$^{-1}$ stock solution were added to the cell suspension to disrupt the cytoplasm completely and eliminate any disturbances due to double-stranded RNA. After at least 30 min incubation at room temperature, samples were analysed using an Ortho 30L Cytofluorograph (Ortho Instruments, Westwood, USA) and the percentages of cells in cell cycle phases were calculated using the method of Krishan and Frei (1976). Each cytofluorographic assay was performed on 20-40 × 10$^3$ cells. Experiments were repeated three times. The coefficient of variation (CV) of the G1 peak of HT29 cells was 4%.

### *Clonogenic assay*

HT29 cells were exposed to graded doses of 5FU (5, 10, 15 μg ml$^{-1}$) for 12 h at 37°C. In combination experiments, cells were pre- or post-treated with 1% NMF for 72 h then exposed to increasing doses of 5FU for 12 h. After exposure, the drug-containing medium was removed, the cells were washed with balanced salt solution, harvested as monodispersed suspension and counted. Known aliquots of cell suspension were seeded in 60-mm Petri dishes into NMF-free medium so that colonies would appear after 14 days of incubation. Colonies were stained with 2% methylene blue in 95% ethanol.

L1210 cells were exposed to graded doses of 5FU (0.1, 0.5, 1.0 μg ml$^{-1}$) for 16 h at 37°C. For the combined treatment,

Correspondence: M. D'Incalci.
Received 3 February 1989; and in revised form 26 July 1989.

after 5FU exposure cells were incubated in growth medium containing 1% NMF for 72 h. The clonogenic ability of treated cells was evaluated by a soft-agar colony assay. Leukaemic colonies were grown by plating the cells in 0.3% agar on the top of a 0.5% agar underlayer. At the end of drug treatment cells were centrifuged, washed in PBS and counted with a haemocytometer. Appropriate dilutions were made to plate 600 viable cells (as determined by erythrosin B dye exclusion test) into 2 $cm^2$ wells of a 24-well plate (Falcon). After 8 days of incubation colonies ($\geqslant$50 cells) were fixed with methanol for 30 s, stained with Mayer's haematoxylin solution for 30 s and counted under a microscope at 10 × magnification.

For the cell lines in combination experiments the NMF-treated cells were taken as control sample and the surviving fraction was calculated by dividing the absolute survival of the treated sample by the absolute survival of the control sample at the same time. Each experimental point was determined in quadruplicate. Experiments were repeated twice.

### *Alkaline elution studies*

To evaluate DNA single strand breaks (SSB) caused by 5FU incorporation in DNA synthesised during drug treatment, exponentially growing HT29 cells were simultaneously exposed to 5FU (1, 5, 10 and 20 $\mu g\ ml^{-1}$) and 0.04 $\mu Ci\ ml^{-1}$ $^{14}C$-thymidine for 16 h. Cells were then washed once in PBS and incubated in NMF-containing and NMF-free medium for 72 h. At different times during this period, alkaline elution experiments were performed according to the method recently reviewed in detail by Kohn *et al.* (1981). Briefly, about $10^6$ cells were resuspended in cold PBS and layered on polycarbonate filters, 0.8 $\mu m$ pore size and 25 mm diameter (Nucleopore Corp., Pleasanton, USA). Cells were then lysed with a solution containing 2% sodium dodecyl sulphate (SDS), 0.02 M $Na_2$ EDTA, 0.1 M glycine, pH 10.0 (lysis solution), which was allowed to flow through the filters by gravity. The outlet of the filter holders was then connected to the pumping system. DNA was eluted from the filters by pumping 0.02 M EDTA solution adjusted to pH 12.6 with tetrapropyl ammonium hydroxide (RSA Corp., Elmsford, USA), containing 0.1% SDS through the filters at 2 ml $h^{-1}$. The pH of the elution buffer was 12.6 instead of the usual 12.1 because at higher pH the alkali labile sites are transformed in DNA SSB during the elution time (Kohn *et al.*, 1981). Three-hour fractions were collected, and fractions and filters processed as described previously (Kohn *et al.*, 1981).

### *DNA, RNA and protein synthesis*

HT29 cells were seeded with 1 × $10^4$ cells $cm^{-2}$ in 35-mm wells of a 6-multiwell culture plate (Falcon) and incubated in a humidified atmosphere for 3 days. After 16 h of exposure to 5 $\mu g\ ml^{-1}$ 5FU cells were allowed to recover in either the absence or the presence of 1% NMF. Control wells were processed by changing the medium at the same time as treated samples. DNA, RNA and protein synthesis were determined by adding radiolabelled precursors at different times after 5FU exposure. The final concentration of each radiolabelled precursor was 1 $\mu Ci\ ml^{-1}$. At the end of radioisotope incubation (1 h) cells were washed once in PBS, harvested and the cell suspension (2 ml) was mixed with 2 ml of cold 10% trichloroacetic acid (TCA) and incubated at 4°C overnight. The precipitate was collected on a 25 mm diameter glass microfibre filter (Whatmann, GF/C) and washed three times with 2 ml cold 5% TCA then twice with 2 ml ethanol. Dried filters were transferred to scintillation vials with 10 ml Pico Fluor TM 15 (Packard) and radioactivity was determined by an LS5800 $\beta$-counter (Beckman Instruments, Irvine, USA). Each point is the average of four replications.

### *In vivo experiments*

Male CD-1 background nu/nu mice (Charles River Laboratories, Calco, Italy), 6–8 weeks old, were employed. They were inoculated in the lateral subcutis site with 2 × $10^6$ cultured HT29 cells and serially transplanted at 4-week intervals. The single cell suspension was obtained from tumours by a standard enzymatic procedure with 0.25% trypsin. Each experimental group consisted of 8–10 animals. Treatment was started when the tumour reached a weight of 120–150 mg (12–15 days post-implantation), according to the following schedules: (a) 5FU 19 mg $kg^{-1}$ $day^{-1}$ for five consecutive days followed by NMF 200 mg $kg^{-1}$ $day^{-1}$ × 10 days; (b) NMF 200 mg $kg^{-1}$ $day^{-1}$ for 10 consecutive days followed by 5FU 19 mg $kg^{-1}$ $day^{-1}$ × 5 days; (c) 5FU 19 mg $kg^{-1}$ $day^{-1}$ × 5 consecutive days followed by 0.9% NaCl × 10 days; (d) 0.9% NaCl × 5 consecutive days followed by NMF 200 mg $kg^{-1}$ $day^{-1}$ × 10 days. All treatments were repeated for two cycles, at 4-day intervals. Mice in control groups received 0.2 ml of 0.9% NaCl solution. 5FU was used at the dose equal to the $LD_0$, and NMF was employed at $<LD_{10}$ assessed in tumour free mice.

The antitumoral effect of the treatments was evaluated in terms of tumour weight inhibition (TWI) and growth delay. Survival data could not be evaluated because tumour-bearing nude mice never die a natural death before their tumour volume and general conditions make euthanasia ethically necessary.

### Results and discussion

Figure 1 illustrates the results of a representative experiment in which HT29 cells were exposed to 5FU for 12 h or to 5FU followed or preceded by 1% NMF for 72 h. NMF had no cytotoxic effect. It is evident that 5FU activity was potentiated only when NMF was given after 5FU. Similar findings have already been reported by this laboratory (Zupi *et al.*, 1988) and were verified also when 5FU exposure was prolonged up to 24 h (data not shown).

A possible explanation for the effect of NMF seen only after 5FU treatment is that this compound prevented the repair of DNA damage that has been reported in cells treated with 5FU (Lonn & Lonn, 1984, 1986; Major *et al.*, 1982). This possibility was also suggested by the observation that NMF reduced the size of the shoulder in the survival curve of HT29 cells. By analogy with survival curves of cells treated with radiation the shoulder could in fact reflect the cells' ability to repair DNA damage caused by the lower doses.

**Figure 1** Dose–response survival curves of HT29 cells exposed to: ○, 5FU alone for 12 h; ●, 5FU for 12 h followed by 1% NMF for 72 h; ■, 1% NMF for 72 h followed by 5FU for 12 h. Per cent survival of cells after 72 h of exposure to NMF was 93 ± 5.6. After incubation with the first agent cells were washed with balanced salt solution and then exposed to the second agent. Where not shown, the standard error is included in symbols.

Using alkaline elution methods we attempted to compare the DNA damage (i.e. DNA breaks plus DNA alkali labile sites) produced by 5FU in HT29 cells and to assess whether the rate of repair of DNA lesions was influenced by post drug-treatment incubation in NMF containing medium. 5FU caused a small number of DNA SSB in HT29 cells (Table I). DNA SSB were only partially repaired at lower concentrations (1 and 5 $\mu$g ml$^{-1}$) and NMF did not appear to influence the repair capacity.

Previous studies indicated that 5FU (Yoshioka *et al.*, 1987) or other thymidylate synthase inhibitors (Lorico *et al.*, 1988) cause DNA double strand breaks which could be important for drug cytotoxicity and that cycloheximide was reducing both the DNA damage and cytotoxicity caused by these compounds. Therefore we investigated whether 5FU or 5FU and NMF were causing DNA double strand breaks by using neutral elution techniques, but the results were negative (data not shown). The fact that NMF potentiates 5FU cytotoxicity without any substantial increase in DNA damage does not bear out the importance of 5FU incorporation into DNA as a crucial part of 5FU activity, at least in HT29 cells.

Since 5FU cytotoxicity could be related to the drug-induced impairment of macromolecule synthesis (Heidelberger, 1965), we investigated whether NMF influenced the alterations of DNA, RNA and protein synthesis in HT29 cells treated with 5FU (Figure 2). NMF alone caused a gradual decline in $^3$H-thymidine incorporation which became evident between 8 and 24 h of exposure. By 48 h of NMF treatment incorporation of the labelled precursor was only 18% of control. In contrast, at the end of 5FU exposure and all through the recovery period $^3$H-thymidine incorporation was considerably increased. Probably cells avidly incorporated $^3$H-thymidine because of depletion of the intracellular thymidine nucleotide pool caused by 5FU-induced inhibition of thymidylate synthase.

Similarly increased incorporation of $^3$H-thymidine was observed in HT29 cells recovering from 5FU exposure in NMF-containing medium, but only up to 24 h of recovery. By 48 h of recovery, $^3$H-thymidine incorporation into DNA was about the same as control cells, probably on account of strong inhibition of DNA synthesis by NMF. As previously reported in murine TLX5 lymphoma cells (Bill *et al.*, 1988), in HT29 human colon carcinoma cells NMF alone causes an arrest of cells in G1 (Table II shows the results of a representative experiment). Probably this blockade does not enhance the cytotoxicity of 5FU (given after NMF), this drug being specific to the S phase. On the other hand, when 5FU is given alone, cells are arrested in S phase and this effect becomes more marked when 5FU treatment is followed by NMF. The NMF-induced inhibition of DNA synthesis, normally reversed without any cytoxocity upon NMF removal, could thus be lethal in a cell whose DNA synthesis capacity has already been impaired by 5FU.

Synthesis of RNA and proteins was not markedly affected by 5FU. NMF reduced RNA synthesis only at later times (48 and 72 h) and this was also seen in HT29 cells pretreated with 5FU. This effect may be a consequence of the inhibition of DNA synthesis that appeared to be more marked and occurred earlier.

The cytotoxic activity of 5FU was not enhanced when

**Table I** DNA single-strand breaks (SSB) caused by 5FU or by 5FU followed by NMF in HT29 cells

| | *5FU ($\mu$g ml$^{-1}$)* | | | |
|---|---|---|---|---|
| | *1* | *5* | *10* | *20* |
| End of treatment[a] | 15–18[b] | 30–38 | 29–42 | 40–56 |
| 24 h recovery without NMF | 5 | 26–33 | 40–47 | 30–33 |
| 24 h recovery with 1% NMF | 4–6 | 16–22 | 33–40 | 21 |
| 48 h recovery without NMF | 4 | 15–17 | 24–28 | 34–35 |
| 48 h recovery with 1% NMF | 5–6 | 15–18 | 40–41 | 32–49 |
| 72 h recovery without NMF | 5–6 | 14–18 | 37–53 | 56–72 |
| 72 h recovery with 1% NMF | 3–4 | 18–20 | 52–68 | 80–90 |

[a]Exposure time 16 h. [b]Range of DNA SSB in rad equivalents.

**Table II** Flow cytometric analysis of cell cycle phase distribution in HT29 cells exposed to 5FU alone, NMF alone or to 5FU followed by NMF

| | *G1* | *SE*[a] | *SM*[a] | *SL + G2M*[a] |
|---|---|---|---|---|
| Control | 58.0 | 10.3 | 10.3 | 21.4 |
| 5FU alone | 26.4 | 34.5 | 23.9 | 15.2 |
| NMF alone | 81.9 | 2.2 | 2.3 | 13.6 |
| 5FU followed by NMF | 13.9 | 42.1 | 26.4 | 17.6 |

HT29 cells were exposed to 0 or 5 $\mu$g ml$^{-1}$ 5FU for 16 h then washed and incubated for 72 h in normal growth medium or in medium containing 1% NMF. The flow cytometric analysis was performed 24 h after 5FU removal. Cell cycle distribution was calculated according to the method of Krishan and Frei (1976). The results of the other two independent experiments were very similar to those reported in the table with maximum variation of 2%.
[a]SE = early S phase; SM = middle S phase; SL = late S phase.

**Figure 2** Evaluation of DNA, RNA and protein synthesis in HT29 cells exposed to 5FU alone (dark columns), to NMF alone (white columns) or to the sequence 5FU to NMF (hatched columns). Cells were seeded and treated with 5 $\mu$g ml$^{-1}$ 5FU for 16 h and then allowed to recover in either the absence or the presence of 1% NMF for 72 h, as described in Materials and methods. To evaluate the effect caused by NMF on macromolecule synthesis, cells were exposed to this agent at the same time as cells which had previously been treated with 5FU. DNA, RNA and protein synthesis were determined adding radiolabelled precursors (methyl-$^3$H-thymidine, 5-$^3$H-uridine, L-4,5,$^3$H-leucine, 1 h at 37°C) at the end of 5FU exposure (time 0) and at different times during the recovery period in NMF-free or NMF-containing medium. Bars indicate the standard error. *$P$ < 0.01, Dunnett's test.

L1210 cells were exposed to NMF (Figure 3) for 72 h after 5FU treatment, suggesting that the synergism is specific for some cell types.

Preliminary results of current investigations in nude mice transplanted with HT29 cells (Table III) indicate that NMF treatment *in vivo* can increase the anti-neoplastic activity of 5FU without raising its bone marrow and general toxicity, thus confirming the data obtained *in vitro*. In fact, only the 5FU to NMF schedule consistently reduced tumour weight (50% TWI) as compared to the reduction produced by the opposite schedule, NMF to 5FU (12% TWI). This finding gains in importance considering that 5FU alone was ineffective against HT29 xenografts. NMF alone had a very slight antitumoral effect (14% TWI). Concerning the NMF's known hepatotoxicity, it is worth noting that in our experimental conditions the different treatment schedules produced very little toxicity. Body weight loss was <10% of the initial weight; haematological analysis and liver histology showed acceptable and completely reversible NMF toxicity.

These results, if confirmed on other human colon tumours, could be of potential clinical interest as a means of increasing the therapeutic efficacy of 5FU in patients with colon cancer.

The generous contribution of the Italian Association for Cancer Research, Milan, Italy, is gratefully acknowledged.

**Figure 3** Dose-response survival curves of L1210 murine leukaemia cells exposed to: O, 5FU for 16 h; ●, 5FU for 16 h followed by 1% NMF for 72 h. Per cent survival of cells after 72 h of exposure to NMF was 115 ± 3.7. After incubation with the first agent cells were washed with PBS and then exposed to the second agent. Where not shown the standard error is included in symbols.

**Table III** Antitumoral effect of different treatment schedules on HT29 human colon cancer xenografts

| *Schedules*[a] | *Tumour weight*[b] *inhibition (% ± s.e.)* | *Tumour growth*[c] *delay (days, median (range)* |
|---|---|---|
| 5FU 19 mg kg$^{-1}$ day$^{-1}$ × 5 days followed by NMF 200 mg kg$^{-1}$ day$^{-1}$ × 10 days | 50[d] ± 20 | 10.5[e] (5–18) |
| NMF 200 mg kg$^{-1}$ day$^{-1}$ × 10 days followed by 5FU 19 mg kg$^{-1}$ day$^{-1}$ × 5 days | 12 ± 5 | 2.5 (1–4) |
| 5FU 19 mg kg$^{-1}$ day$^{-1}$ × 5 days followed by 0.9% NaCl × 10 days | – | 0.5 (0–1.5) |
| 0.9% NaCl × 5 days followed by NMF 200 mg kg$^{-1}$ day$^{-1}$ × 10 days | 14 ± 5.5 | 1.0 (0–2) |

[a]All treatments were repeated for two cycles, at 4-day intervals; [b]Tumour weights at the end of the second cycle of treatment; [c]Time to reach 2 × $10^3$ mg; [d]Statistically different ($P$ <0.05) from values of controls or of other groups as assessed by Dunnett test; [e]Statistically different ($P$ <0.01) from values of controls or of other groups as assessed by Mann–Whitney test.

### References

BILL, C.A., GESCHER, A. & HICKMAN, J.A. (1988). Effects of N-methylformamide on the growth, cell cycle and glutathione status of murine TLX5 lymphoma cells. *Cancer Res.*, **48**, 3389.

DEXTER, D.L., LEE, E.S., BLIVEN, S.F., GLICKSMAN, A.S. & LEITH, J.T. (1984). Enhancement by N-methylformamide of the effect of ionizing radiation on a human colon tumor xenografted in nude mice. *Cancer Res.*, **44**, 4942.

HARPUR, E.S., LANGDON, S.P., FATHALLA, S.A.K. & ISHMAEL, J. (1986). The antitumour effect and toxicity of cis-platinum and *N*-methylformamide in combination. *Cancer Chemother. Pharmacol.*, **16**, 139.

HEIDELBERGER, C. (1965). Fluorinated pyrimidines. In *Progress in Nucleic Acid Research and Molecular Biology, vol. 4*, Davidson, J.N. & Cohn, W.E. (eds) p. 1. Academic Press: New York.

KOHN, K.W., EWING, R.A.G., ERICKSON, L.C. & ZWELLING, L.A. (1981). Measurement of strand breaks and cross-links by alkaline elution. In *DNA Repair: a Laboratory Manual of Research Procedures, vol. 1 part B*, Friedberg, E.C. & Hanawalt, P.C. (eds) p. 379. Marcel Dekker: New York.

KRISHAN, A. & FREI, E. III (1976). Effect of adriamycin on the cell cycle traverse and kinetics of cultured human lymphoblasts. *Cancer Res.*, **36**, 143.

LANGDON, S.P., HICKMAN, J.A., GESCHER, A., STEVENS, M.F.G., CHUBB, D. & VICKERS, L.M. (1985). *N*-Methylformamide (NSC 3051): a potential candidate for combination chemotherapy. *Eur. J. Cancer Clin. Oncol.*, **21**, 745.

LANGDON, S.P. & HICKMAN, J.A. (1987). Alkylformamides as inducers of tumour cell differentiation. A mini review. *Toxicology*, **43**, 239.

LEITH, J.T., LEE, E.S., LEITE, D.V. & GLICKSMAN, A.S. (1986). Enhanced x ray sensitivity of human colon tumor cells by combination of *N*-methylformamide with chemotherapeutic agents. *Int. J. Radiat. Oncol. Biol. Phys.*, **12**, 1423.

LEITH, J.T., LEE, E.S., VAYER, A.J. Jr., DEXTER, D.L. & GLICKSMAN, A.S. (1985). Enhancement of the responses of human colon adenocarcinoma cells to x-irradiation and cis-platinum by *N*-methylformamide (NMF). *Int. J. Radiat. Oncol. Biol. Phys.*, **11**, 1971.

LONN, U. & LONN, S. (1984). Interaction between 5-fluorouracil and DNA of human colon adenocarcinoma. *Cancer Res.*, **44**, 3414.

LONN, U. & LONN, S. (1986). DNA lesions in human neoplastic cells and cytotoxicity of 5-fluoropyrimidines. *Cancer Res.*, **46**, 3866.

LORICO, A., TOFFOLI, G., BOIOCCHI, M. & 4 others (1988). Accumulation of DNA strand breaks in cells exposed to methotrexate or *N*10-propargyl-5,8-dideazafolic acid. *Cancer Res.*, **48**, 2036.

LOTAN, R. & NICOLSON, G.L. (1988). Can anticancer therapy be improved by sequential use of cytotoxic and cytostatic (differentiating or immunomodulating) agents to suppress tumor cell phenotypic diversification? *Biochem. Pharmacol.*, **37**, 149.

MAJOR, P.P., EGAN, E., HERRICK, D. & KUFE, D.W. (1982). 5-Fluorouracil incorporation in DNA of human breast carcinoma cells. *Cancer Res.*, **42,** 3005.

YOSHIOKA, A., TANAKA, S., HIRAOKA, O. & 6 others (1987). Deoxyribonucleoside triphosphate imbalance. 5-Fluorodeoxyuridine-induced DNA double strand breaks in mouse FM3A cells and the mechanism of cell death. *J. Biol. Chem.*, **262,** 8235.

ZUPI, G., MARANGOLO, M., ARANCIA, G. & 5 others (1988). Modulation of the cytotoxic effect of 5-fluorouracil by *N*-methylformamide on a human colon carcinoma cell line. *Cancer Res.*, **48,** 6193.


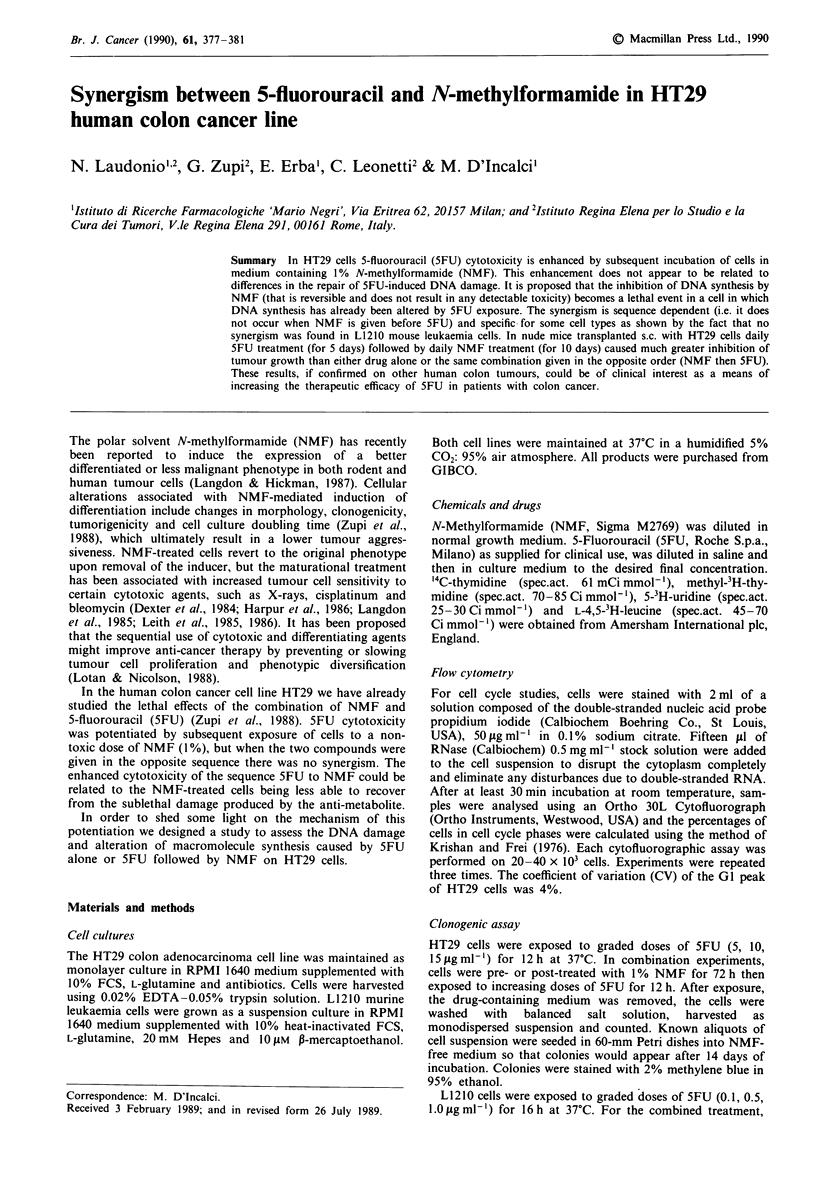

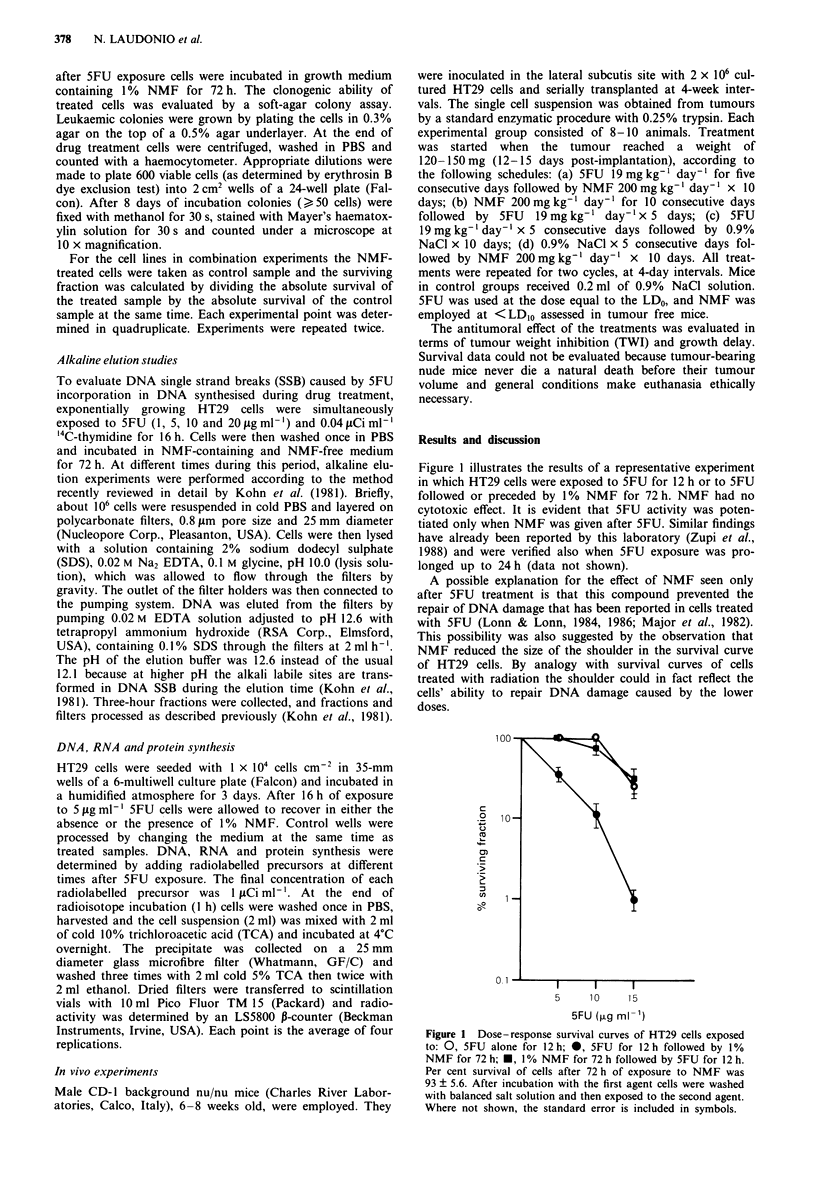

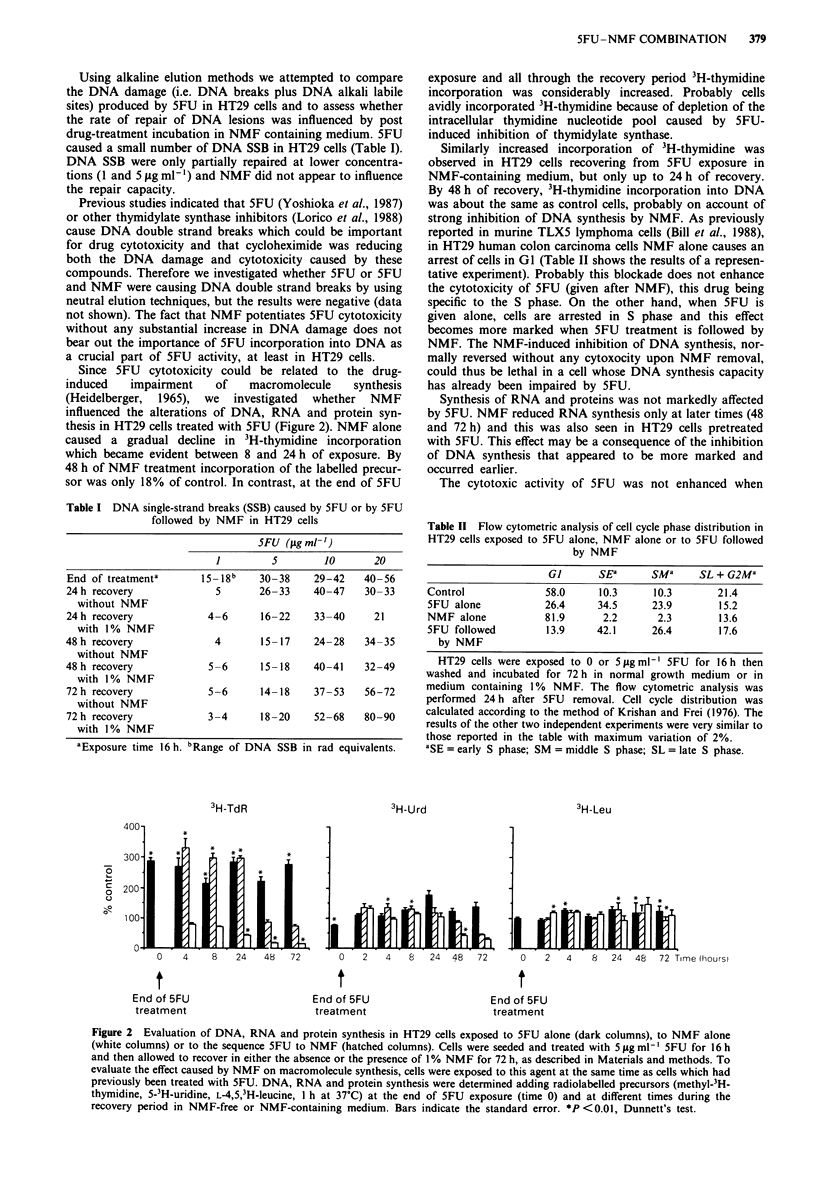

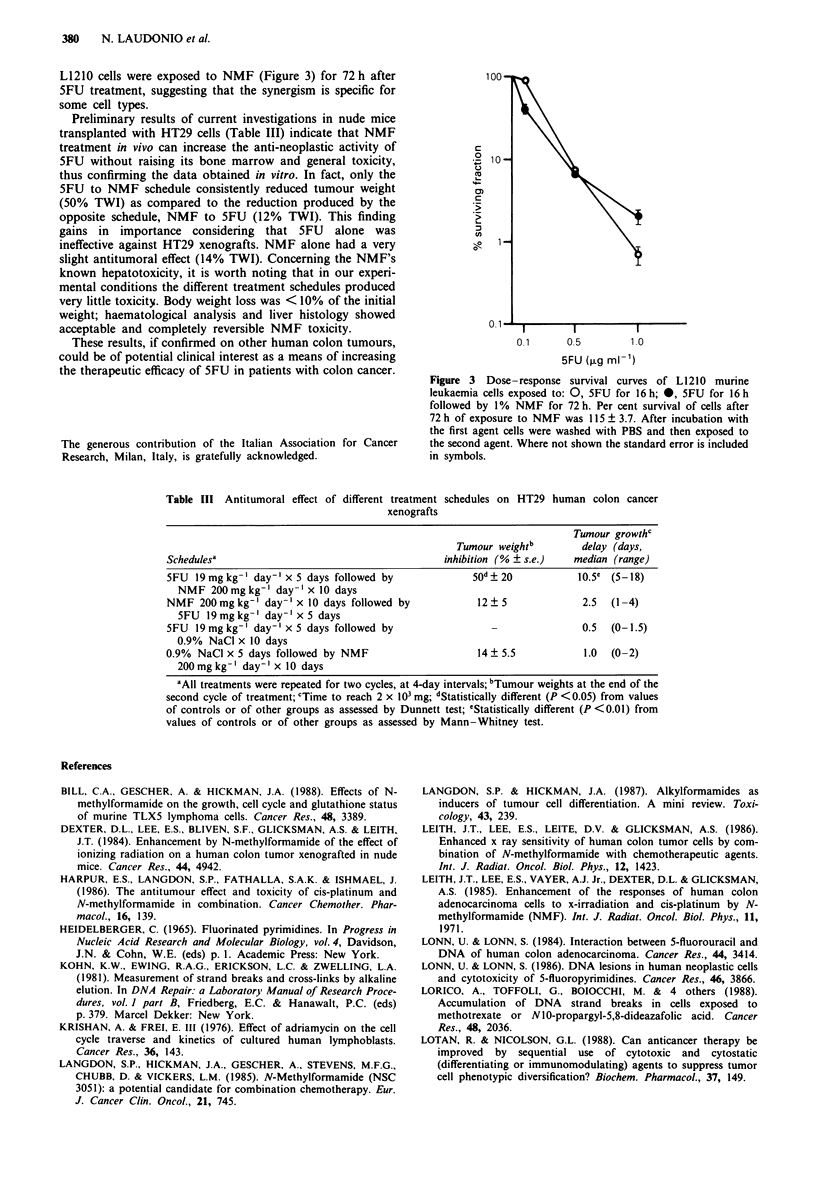

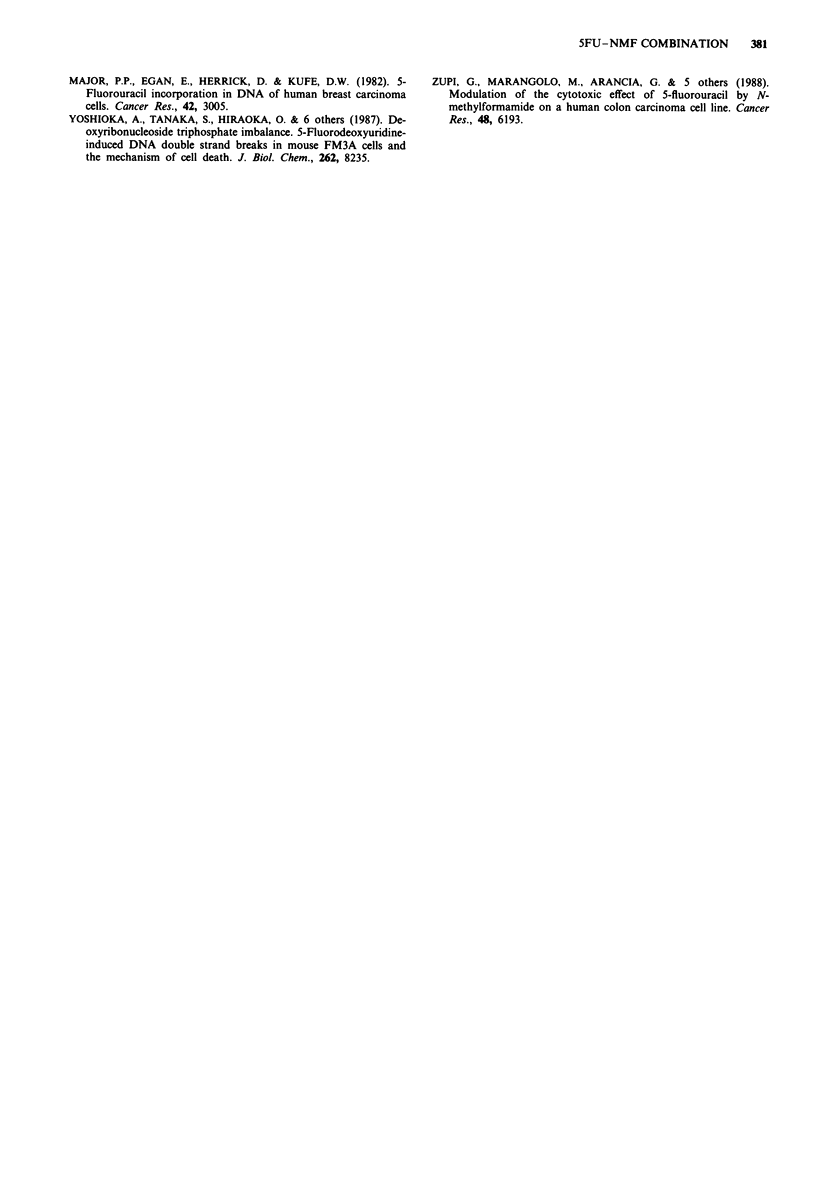


## References

[OCR_00545] Bill C. A., Gescher A., Hickman J. A. (1988). Effects of N-methylformamide on the growth, cell cycle, and glutathione status of murine TLX5 lymphoma cells.. Cancer Res.

[OCR_00550] Dexter D. L., Lee E. S., Bliven S. F., Glicksman A. S., Leith J. T. (1984). Enhancement by N-methylformamide of the effect of ionizing radiation on a human colon tumor xenografted in nude mice.. Cancer Res.

[OCR_00556] Harpur E. S., Langdon S. P., Fathalla S. A., Ishmael J. (1986). The antitumour effect and toxicity of cis-platinum and N-methylformamide in combination.. Cancer Chemother Pharmacol.

[OCR_00574] Krishan A., Frei E. (1976). Effect of adriamycin on the cell cycle traverse and kinetics of cultured human lymphoblasts.. Cancer Res.

[OCR_00585] Langdon S. P., Hickman J. A. (1987). Alkylformamides as inducers of tumour cell differentiation--a mini-review.. Toxicology.

[OCR_00579] Langdon S. P., Hickman J. A., Gescher A., Stevens M. F., Chubb D., Vickers L. M. (1985). N-Methylformamide (NSC 3051): a potential candidate for combination chemotherapy.. Eur J Cancer Clin Oncol.

[OCR_00590] Leith J. T., Lee E. S., Leite D. V., Glicksman A. S. (1986). Enhanced X ray sensitivity of human colon tumor cells by combination of N-methylformamide with chemotherapeutic agents.. Int J Radiat Oncol Biol Phys.

[OCR_00596] Leith J. T., Lee E. S., Vayer A. J., Dexter D. L., Glicksman A. S. (1985). Enhancement of the responses of human colon adenocarcinoma cells to X-irradiation and cis-platinum by N-methylformamide (NMF).. Int J Radiat Oncol Biol Phys.

[OCR_00609] Lorico A., Toffoli G., Boiocchi M., Erba E., Broggini M., Rappa G., D'Incalci M. (1988). Accumulation of DNA strand breaks in cells exposed to methotrexate or N10-propargyl-5,8-dideazafolic acid.. Cancer Res.

[OCR_00615] Lotan R., Nicolson G. L. (1988). Can anticancer therapy be improved by sequential use of cytotoxic and cytostatic (differentiating or immunomodulating) agents to suppress tumor cell phenotypic diversification?. Biochem Pharmacol.

[OCR_00606] Lönn U., Lönn S. (1986). DNA lesions in human neoplastic cells and cytotoxicity of 5-fluoropyrimidines.. Cancer Res.

[OCR_00603] Lönn U., Lönn S. (1984). Interaction between 5-fluorouracil and DNA of human colon adenocarcinoma.. Cancer Res.

[OCR_00623] Major P. P., Egan E., Herrick D., Kufe D. W. (1982). 5-Fluorouracil incorporation in DNA of human breast carcinoma cells.. Cancer Res.

[OCR_00628] Yoshioka A., Tanaka S., Hiraoka O., Koyama Y., Hirota Y., Ayusawa D., Seno T., Garrett C., Wataya Y. (1987). Deoxyribonucleoside triphosphate imbalance. 5-Fluorodeoxyuridine-induced DNA double strand breaks in mouse FM3A cells and the mechanism of cell death.. J Biol Chem.

[OCR_00634] Zupi G., Marangolo M., Arancia G., Greco C., Laudonio N., Iosi F., Formisano G., Malorni W. (1988). Modulation of the cytotoxic effect of 5-fluorouracil by N-methylformamide on a human colon carcinoma cell line.. Cancer Res.

